# Commentary: An Even Clearer Portrait of Bias in Observational Studies?

**DOI:** 10.1097/EDE.0000000000000302

**Published:** 2015-06-02

**Authors:** Neil M. Davies

**Affiliations:** From the a Medical Research Council Integrative Epidemiology Unit, University of Bristol, Bristol, United Kingdom; and b School of Social and Community Medicine, University of Bristol, Bristol, United Kingdom.

Instrumental variable analysis is an increasingly popular statistical method in epidemiologic research.^1^ Epidemiologists’ enthusiasm for this approach may be because it can potentially estimate causal effects in observational data in the presence of unmeasured confounding.^2^ This overcomes a significant limitation of conventional epidemiologic methods, such as multivariable regression analysis: residual confounding. However, it is also possible for instrumental variable analyses to suffer from residual confounding. This can occur if the proposed instruments are associated with unmeasured confounding factors. Therefore, the key question for empirical researchers, regulators, and clinicians is: which is more biased—conventional multivariable adjusted regression or instrumental variable analysis?

In this issue of Epidemiology, Jackson and Swanson^3^ elegantly describe a method for presenting and comparing the balance of potential confounders across values of the instrument and the actual treatment. This can allow researchers to assess the relative bias that could be caused by observed confounding factors. These methods may provide information about the relative bias of the unobserved confounders if they are correlated with the observed confounders. I will briefly discuss the methodologic improvements proposed by this article, its limitations, and finally a potential solution to these limitations.

## A BRIEF DESCRIPTION OF THE MAIN RESULTS

The core of the paper is illustrated by a standard linear model:


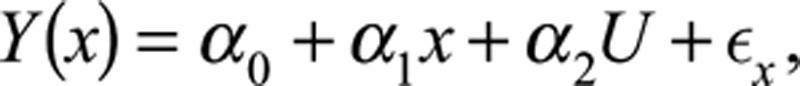


where *Y*, *x*, *U*, and *z*, respectively, represent a binary outcome, the treatment, a confounder of the outcome-treatment relationship, and the instrument. ϵ_x_ represents the error term, *α*_0_ is a constant, *α*_1_ is the effects of the treatment, and *α*_2_ is the effects of the confounder on the outcome.

The simplest approach for assessing bias is to compare the difference in each confounder across values of the actual treatment, *x*, to the difference in the confounder across values of the instrument z. However, the same difference in a confounder across values of the instrument will result in much larger bias in the instrumental variable estimator than for the ordinary least squares (OLS) estimator.

To overcome this, Brookhart and Schneeweiss^4^ proposed reporting the relative bias of the OLS and instrumental variable estimators of *α*_1_ when the confounder U is omitted.

The OLS bias is


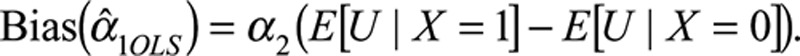


The instrumental variable bias is


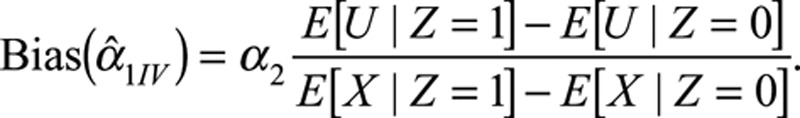


These biases can be compared by computing the ratio of covariate imbalance across the actual treatment and instrument:


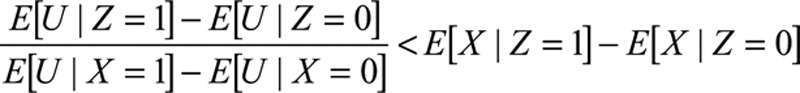


If the ratio on the left (known as the prevalence difference ratio) is larger than the term on the right (the strength of the instrument), then the instrumental variable bias is likely to be larger than the OLS bias.

Despite this approach’s relative simplicity, it has been rarely used in the literature. Furthermore, Jackson and Swanson^3^note some limitations to prevalence difference ratios—they only provide information about relative bias and provide no information about the absolute bias. This means that if the instrumental variable and OLS biases are both very small a researcher could still find a very large prevalence difference ratio if the instrumental variable bias is small, but slightly larger than the OLS bias.

Jackson and Swanson^3^ suggest presenting the bias components in graphical form. The effect of the confounder on the outcome, *α*_2_, is the same for both the instrumental variable and OLS estimators, so any difference in bias between the two approaches must be due to the difference between the right hand terms in the bias equations. Therefore, it is sufficient to compare:


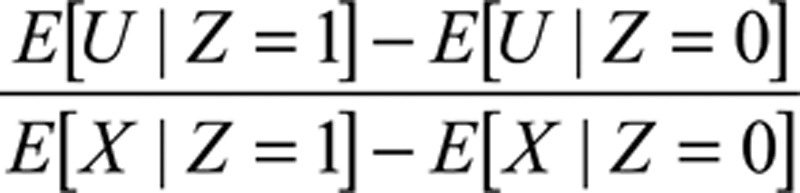


to


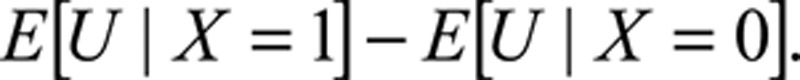


## LIMITATIONS

The major limitation of this approach is that the authors do not propose any methods for estimating the standard errors of the instrumental variable bias and the OLS bias. As with instrumental variable estimates of the effects of the treatment, the scaled instrumental variable bias components will have much larger standard errors and confidence intervals than the OLS bias. This means that, solely by chance, across a set of potential confounding factors the scaled instrumental variable bias components are likely to be much larger for some covariates. However, these differences may simply reflect statistical noise. To overcome this, for each covariate, we need to test the null hypothesis that there are no differences between instrumental variable and the OLS biases.

## A SIMPLE SOLUTION TO THESE LIMITATIONS

A simple way to overcome this limitation is to note that the two bias terms are equivalent to the instrumental variable estimate (a Wald estimator) of the effects of the treatment *x* on the confounder, and the OLS estimate of the association of the treatment and the confounder. Recapitulating these results using standard instrumental variable estimation methods allows researchers to estimate the bias terms using existing packages, such as reg and ivreg2 in Stata.^5,6^ This allows us to estimate the confidence intervals of the bias terms. These confidence intervals can be added to the covariates balance plots. Furthermore, within this framework, we can test the null hypothesis of no differences between the OLS and instrumental variables biases using Hausman tests.^7^

## EMPIRICAL ILLUSTRATION

To illustrate the benefits of this approach, I reanalyzed the results of my paper investigating the relative effects of paroxetine versus other selective serotonin reuptake inhibitors (SSRIs) on self-harm and suicide.^8^ The instrumental variable is the patient’s physician’s preferences for paroxetine or another SSRI. This is unmeasured, so we used the physicians’ previously prescribed prescriptions as a proxy for their preferences. Brookhart et al.^9^ argued that physicians’ preferences for medications were plausible instruments because they are related to the medications they issue and may not be related to patient-level confounding factors. Please see the full paper for details of the sample and methods. I previously reported that the prevalence difference ratios for six of the 12 covariates suggested that the instrumental variable bias was larger than the OLS bias (Table 4 of the referenced paper). In Table, I report (1) the estimates of the OLS bias of the actual treatment (equal to one if the patient was prescribed paroxetine zero otherwise) and each of the 12 covariates, (2) the estimates of instrumental variables bias, and (3) Hausman tests of the difference between the estimated biases.

**TABLE.**
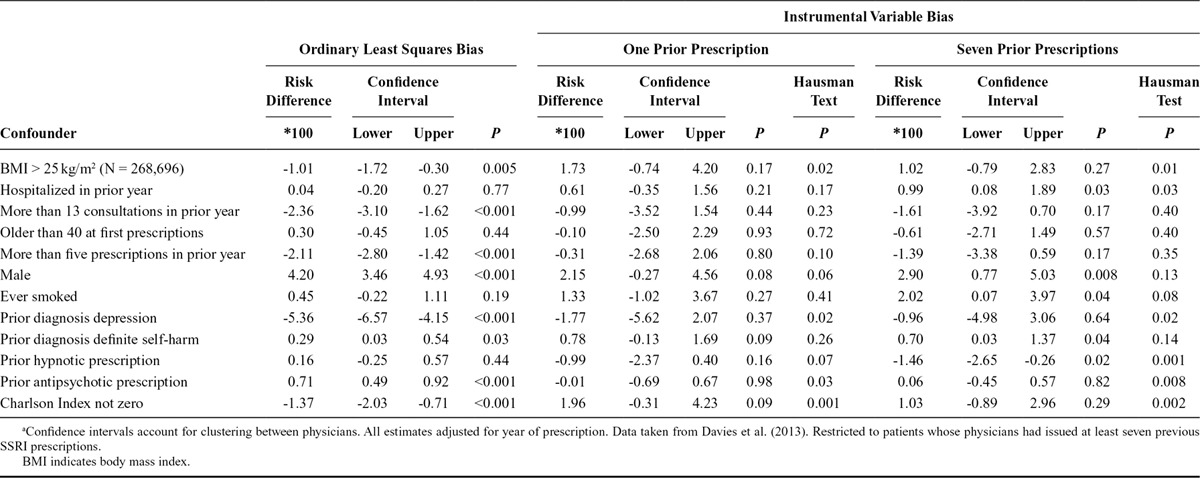
Ordinary Least Squares and Instrumental Variable Bias When Comparing Paroxetine and Other SSRIs (N = 359,736)^a^

I found evidence that patients prescribed paroxetine were different to those prescribed other SSRIs for eight of the 12 covariates. The instrumental variable biases were much less precise, but there was weak evidence of differences for four of the 12 covariates by values of the instrument. The differences between the OLS and instrumental variable biases, as indicated by the Hausman tests, were substantial. For six of the 12 covariates these tests suggested that the instrumental variable bias was either smaller or in the opposite direction to the OLS bias. The importance of presenting confidence intervals can clearly be seen in the Figure. If only the point estimates were presented, we might erroneously conclude that the instrumental variable bias is larger for six of 12 covariates. However, we can only reject the Hausman test for two of differences (body mass index and Charlson index), and for these covariates the instrumental variable and OLS biases are opposite directions.

**FIGURE.**
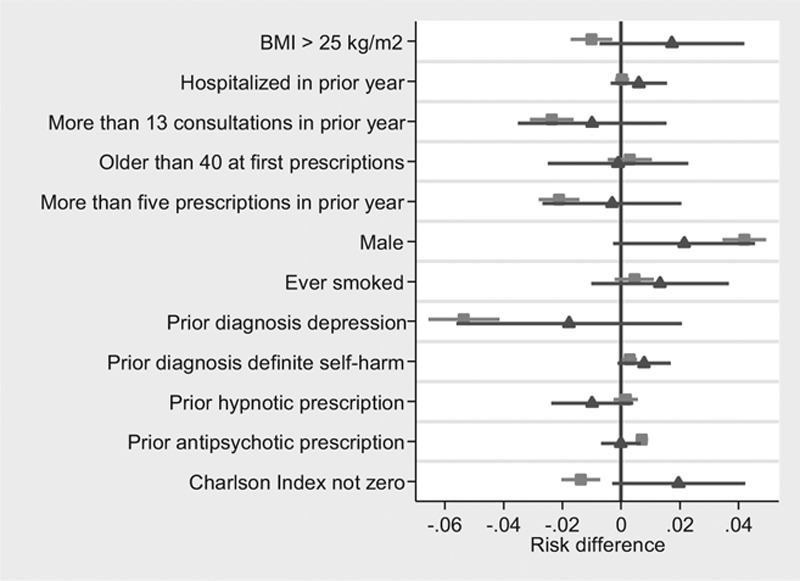
Covariate balance by levels of treatment (*squares*) and levels of the proposed instrument (*triangles*) using individual level data published by Davies et al.^7^ (N = 359,736). Notes: Covariates binary variables, robust standard errors clustered by physician.

Two further advantages of using a standard instrumental variable framework to estimate the bias terms is that it is generalizable to multiple instrument settings, and we can test for differences in the biases between different sets of instruments using Hansen tests.^10^

## CONCLUSIONS, ONGOING WORK, AND SUGGESTIONS FOR FUTURE RESEARCH

Jackson and Swanson^3^ make an important contribution to the literature. When comparing OLS and instrumental variable biases, researchers should account for the instrument strength. However, these graphical specification tests are even clearer if researchers present confidence intervals and test hypotheses. These tests are simple to compute and should be reported by studies using instrumental variable analysis in pharmacoepidemiology and genetic epidemiology. These techniques are a welcome addition to a rapidly growing literature describing and critiquing the use of instrumental variable methods.^11–15^ I fully expect that these and other developments will overcome some of the known limitations of instrumental variable methods. And perhaps more importantly, researchers will precisely describe situations when instrumental variable analysis is likely to be more biased. Only if this is accomplished will epidemiologists be in a position to credibly advise regulators, clinicians, and patients about whether instrumental variable analysis or conventional multivariable regression gives the least biased indication of causal treatment effects.

## ABOUT THE AUTHOR

NEIL DAVIES is a Research Associate at the MRC Integrative Epidemiology Unit at the University of Bristol. He has published a number of studies that have used instrumental variable methods in genetic epidemiology and pharmacoepidemiology.
